# Lower protein expression levels of MHC class I chain-related gene A in hepatocellular carcinoma are at high risk of recurrence after surgical resection

**DOI:** 10.1038/s41598-018-34155-7

**Published:** 2018-10-25

**Authors:** Chung-Feng Huang, Shu-Chi Wang, Wen-Tsan Chang, Ming-Lun Yeh, Ching-I Huang, Zu-Yau Lin, Shinn-Cherng Chen, Wan-Long Chuang, Jee-Fu Huang, Chia-Yen Dai, Yao-Li Chen, Ming-Lung Yu

**Affiliations:** 1 Hepatobiliary Division, Department of Internal Medicine, Kaohsiung Medical University Hospital, Kaohsiung Medical University, Kaohsiung, Taiwan; 2 Faculty of Internal Medicine, School of Medicine, College of Medicine, Kaohsiung Medical University, Kaohsiung, Taiwan; 3 Department of Occupational Medicine, Kaohsiung Medical University Hospital, Kaohsiung Medical University, Kaohsiung, Taiwan; 4 Department of Medical Laboratory Science and Biotechnology, Kaohsiung Medical University, Kaohsiung, Taiwan; 5 Division of General and Digestive Surgery, Department of Surgery, Kaohsiung Medical University Hospital, Kaohsiung, Taiwan; 6 Faculty of Surgery, School of Medicine, College of Medicine, Kaohsiung Medical University, Kaohsiung, Taiwan; 7 Department of Preventive Medicine, Kaohsiung Medical University Hospital, Kaohsiung Medical University, Kaohsiung, Taiwan; 8 Division of General Surgery, Department of Surgery, Changhua Christian Hospital, Changhua, Taiwan; 9 Institute of Biomedical Sciences, National Sun Yat-Sen University, Kaohsiung, Taiwan; 10 College of Biological Science and Technology, National Chiao Tung University, Hsin-Chu, Taiwan

## Abstract

MHC class I chain-related gene A (MICA) variants have been associated with hepatocellular carcinoma (HCC). Their association with MICA expression in cancer cells and cancer recurrence is unknown. SNP rs2596542 of MICA was tested in 193 HCC patients with surgical resection. The corresponding MICA expression in the cancer tissue was measured by immunochemistry microarray. Patients with the SNP rs2596542 A allele had significantly lower MICA expression in tumor tissue than did those with the GG genotype (24.7 ± 15.1% vs. 41.5 ± 23.4%, P < 0.001). Patients who had HCC recurrence had significantly lower MICA expression in tumor tissue (34.2 ± 21.8% vs. 24.0 ± 19.8%, P = 0.03). Cox regression analysis revealed that the factors independently predictive of HCC recurrence included low MICA expression (hazard ratio [HR]/95%confidence intervals [CI]: 2.77/1.07–7.14, P = 0.035) and tumor size (HR/CI: 5.22/2.11–12.96, P < 0.001). Compared to patients with tumors <5 cm and MICA expression >30%, patients with either one and both two risk factors had HCC HRs of 9.76 (C.I. 1.27–75.03, P = 0.03) and 27.30 (C.I. 3.46–215.6, P = 0.002), respectively. We concluded that low cellular MICA expressions were at a greater risk of HCC recurrence after curative treatment.

## Introduction

Liver cancer is the second leading cause of cancer mortality worldwide^1^. Hepatocellular carcinoma (HCC) is the most common (>90%) primary malignancy of the liver. It is estimated that more than 0.7 million die of HCC each year, and drastically increasing annual death rates have been observed over the past two decades^2–4^. Therefore, the disease burden of HCC remains a major threat public health. The occurrence of HCC is stepwise and multifactorial. Viral hepatitis, alcohol consumption, diabetes and environmental triggers (ex. aflatoxin) account for the major causes. By contrast, host genetic predispositions have been recognized as additional potential causes of HCC. Genome-wide association studies (GWAS) have extensively explored host genomes associated with HCC at the single-nucleotide level. Several studies using GWAS have identified certain single nucleotide polymorphisms (SNPs) that are associated with hepatocarcinogenesis^5–7^. Among them, the SNP rs2596542 of MHC class I chain-related gene A (MICA) has been linked to HCC susceptibility in patients with hepatitis B virus (HBV) and HCV infection. Genetic predisposition may contribute to HCC occurrence with variable impacts depending on different etiologies and ethnicities^7–9^.

MICA is the ligand for the natural killer cell group 2D receptor (NKG2D). Overproduction of soluble MICA in the circulation may down-regulate NKG2D expression in immune cells, further diminishing NKG2D-mediated anti-tumor immunity. Previous studies have demonstrated a link between an elevated serum MICA level and HCC risk^7,9–11^. Notably, MICA is a transmembrane protein, and the expression of cellular MICA in HCC patients in the clinical setting has rarely been explored. No studies have investigated whether the expression of cellular MICA differs among Asian HCC patients with different MICA genetic predispositions. In this study, we recruited a large cohort of well-characterized HCC patients who received curative surgical resection and post-operative follow-up. We aimed to study the effects of the host genetics of MICA and MICA expression on the cancer tissues. We further sought to elucidate the association of MICA expression in the cancer tissues with patient prognosis in terms of HCC recurrence.

## Methods

HCC patients who underwent surgical resection were consecutively enrolled from 2010 to 2014. HCC was confirmed by histology or clinical diagnosis based on the guidelines of the American Association for the Study of Liver Diseases^3^ and the Asian Pacific Association for the Study of the Liver^4^. The clinical characteristics of the patients, including gender, age, HCC etiology, underlying liver fibrosis, cancer stage, tumor size and tumor number, were collected for further analysis. The cancer stage was evaluated based on the American Joint Committee on Cancer (AJCC), 7^th^ edition TNM staging system^12^ and the Barcelona Clinic Liver Cancer (BCLC) staging classification^13^. All patients received regular surveillance for HCC recurrence after surgery. The institutional review board of the Kaohsiung Medical University Hospital approved the protocols (KMUHIRB-20130101), which conformed to the guidelines of the International Conference on Harmonization for Good Clinical Practice. All patients provided written informed consent. All procedures followed were by the ethical standards of the responsible committee on human experimentation and with the Helsinki Declaration of 1975, as revised in 2008.

### Tissue array preparation and immunochemistry staining of MICA in the cancer tissues

MICA expression in the surgical tissues was measured by immunochemistry staining^14,15^. Liver samples including tumor tissue and peritumoral tissue were embedded in formalin-fixed paraffin. A clear-cut hematoxylin-stained section corresponding to the paraffin block was selected for tissue microarray template designation. A 4 mm thickened tissue array block was cut thereafter for the immunochemistry stain.

The expression patterns of the MICA protein were detected by the BIOTnA IHC kit. Briefly, incubated sections were treated with a primary mouse anti-MICA antibody (1:50 dilution; clone number: 159207, R&D system, USA) in antibody diluent (TA00D, BIOTnA) followed by rabbit/mouse HRP label (TAHC03 IHC test sample, BIOTnA) detection reagent. H&E stain was used for nuclear staining. After the staining procedures, the tissue sections were mounted and observed. The samples were observed by an optical microscope and analyzed by Image-Pro Plus 6.0 to count the percentage of the positive cells that were brown.

### MICA rs2596542 genetic testing

The SNP rs2596542 of MICA was determined by ABI TaqMan^®^ SNP genotyping assays (Applied Biosystems, Foster City, CA, USA) using pre-designed, commercial genotyping assays (ABI Assay ID: C__27301153_10)^9,16^. Briefly, PCR primers and two allele-specific probes were designed to detect a specific SNP target. PCR reactions were performed in 96-well microplates with the ABI 7500 real-time PCR system. Allele discrimination was achieved by detecting fluorescence using System SDS software version 1.2.3. The allele and genotype frequencies were consistent with Hardy-Weinberg equilibrium.

#### Statistical analyses

The frequency was compared between groups using the χ^2^ test with the Yates correction or using Fisher’s exact test. Group means were compared using analysis of variance and Student’s *t* test or the Mann-Whitney U test. The MICA SNP was calculated using a dominant (genotype GG vs. AG/AA) genetic model of inheritance. Statistical correlations between MICA expression in the tumor tissue and the peritumoral tissue were determined by Spearman’s test. Linear regression analysis was performed to determine the factors correlated with MICA expression in the tumor. The area under the curve (AUC) was compared using receiver operating characteristic (ROC) analysis to determine the cut-off value for the expression of MICA in patients with different MICA genetic variants. A stepwise logistic regression analysis was performed to evaluate the independent factors associated with high levels of MICA expression in the tumor tissue. To avoid the issue of incomplete resection, patients with recurrent HCC within six months after surgery were excluded when judging the factors predictive of HCC recurrence^17^. Kaplan–Meier analysis and the log-rank test were performed by comparing the differences in HCC recurrence between the determining factors. The risk factors independently associated with HCC recurrence were evaluated using Cox regression analysis. The statistical analyses were performed using the SPSS 12.0 statistical package (SPSS, Chicago, IL, USA). All statistical analyses were based on two-tailed hypothesis tests with a significance level of P < 0.05.

## Results

### Patient characteristics

A total of 193 HCC patients who underwent surgical resection were enrolled for analysis. The mean age was 62.8 years, and males accounted for 74.6% (n = 144) of the population. Of the 187 patients with available MICA SNP, ninety (46.6%) patients carried the MICA rs2596542 A allele (Table 1).

**Table 1:** Characteristics of the 193 HCC patients.

Age (years, mean ± SD)	62.8 ± 11.5
Male gender, n (%)	144 (74.6)
BMI (kg/m^2^, mean ± SD)	24.9 ± 4.4
AST (IU/L, mean ± SD)	56 ± 38
ALT (IU/L, mean ± SD)	51 ± 40
α-fetoprotein >20 ng/mL, n (%)	83 (43.0)
Fibrosis stage 3–4, n (%)	104 (53.9)
HBV/HCV/B + C/NBNC/unknown, n	87/51/7/37/11
Child-Pugh score A, n (%)	163 (84.5)
MICA rs2596542 A allele, n (%)*	90 (46.6)
BCLC stage 0-A, n (%)	117 (60.6)
TMN stage 1-2, n (%)	159 (82.4)
Tumor size >5 cm, n (%)	70 (36.3)
Tumor number >2, n (%)	32 (16.6)
Poor differentiated HCC, n (%)^†^	48 (40.3)

Note: BMI: body mass index.AST: aspartate aminotransferase. ALT: alanine aminotransferase. HBV: hepatitis B virus. HCV: hepatitis C virus. B + C: hepatitis B and hepatitis C coinfection. NBNC: non-hepatitis B and non-hepatitis C. HCC: hepatocellular carcinoma. BCLC: Barcelona Clinic Liver Cancer. MICA: MHC class I chain-related A. ^*^6 patients had indeterminate single nucleotide polymorphism. ^†^Data available in 119 patients.

### Expression level of cellular MICA and the MICA rs2596542 polymorphism

The level of MICA in the tumor tissue and the peritumoral tissue was 33.1 ± 21.4% and 30.4 ± 16.9%, respectively (P = 0.18). The MICA expression in the tumor tissue correlated well to that in the peritumoral tissue (P < 0.001, *r* = 0.364).

**Figure 1:**
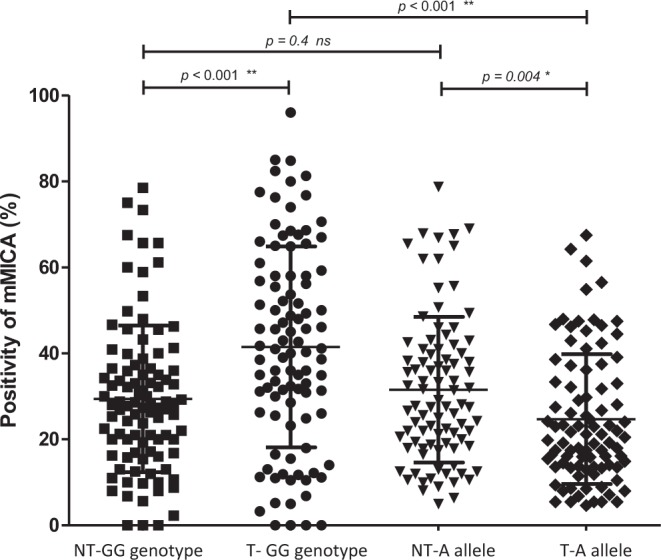
The expression of cellular MICA in patients with different MICA rs2596542 genotypes. T: tumor tissue. NT: non-tumor part of the tissue. A allele: MICA rs2596542 allele. GG genotype: MICA rs2596542 GG genotype.

**Figure 2:**
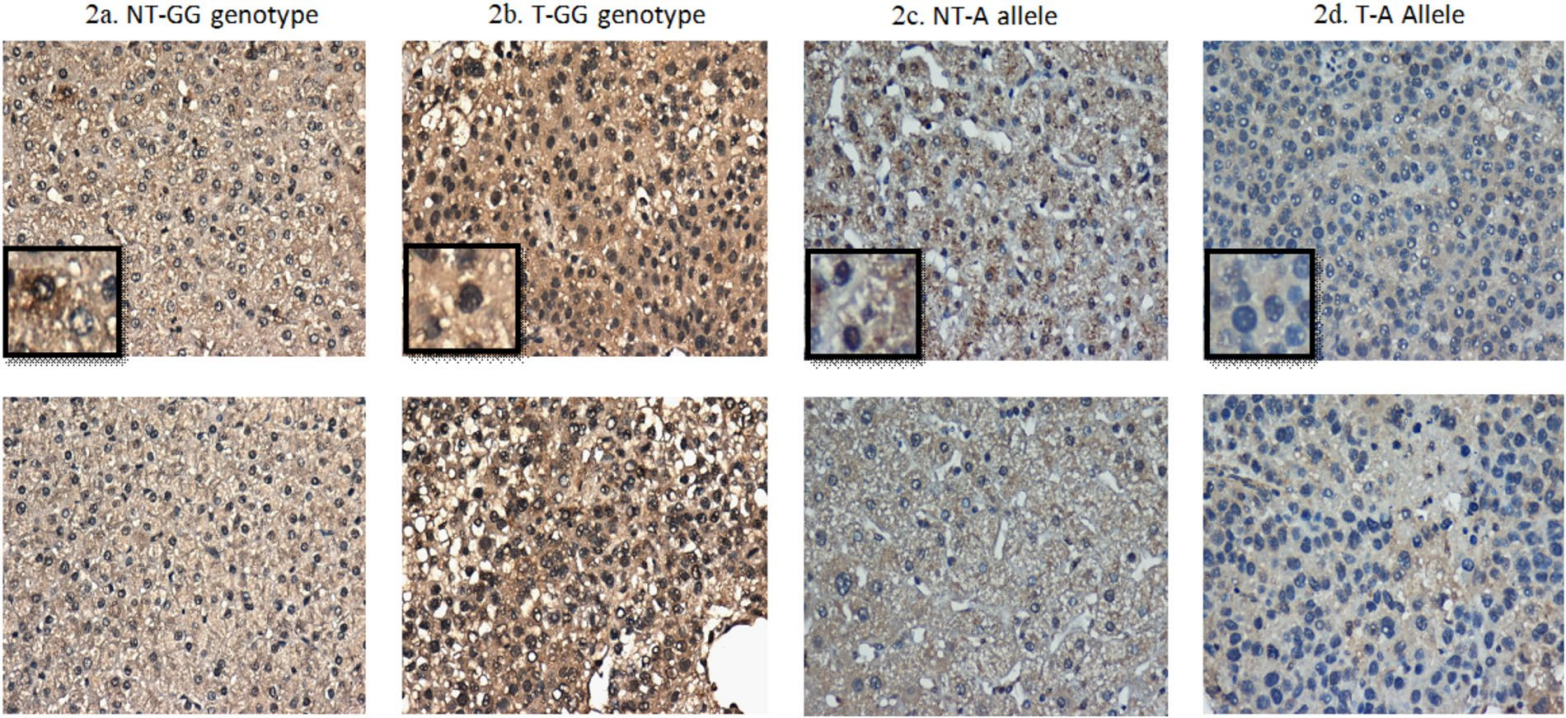
The areas with brown color indicate the presentation of cellular MICA. T: tumor tissue. NT: non-tumor part of the tissue. A allele: MICA rs2596542 allele. GG genotype: MICA rs2596542 GG genotype.

We further analyzed the MICA expression levels in the tumor tissue and peritumoral tissue between patients with different MICA genetic variants (Figs 1 and 2). Among patients with the GG genotype, the MICA expression levels were significantly higher in the tumor tissue than in the peritumoral tissue (41.5 ± 23.4% vs. 29.4 ± 17.0%, P < 0.001). By contrast, among A allele carriers the MICA expression levels were significantly lower in the tumor tissue than in the peritumoral tissue (24.7 ± 15.1% vs. 31.5 ± 16.9%, P = 0.005). Interestingly, the MICA expression levels in the peritumoral tissue were similar between patients with the MICA rs2596542 A allele and those with the GG genotype (31.5 ± 16.9% vs. 29.4 ± 17.0%, P = 0.39). In contrast, the MICA expression levels in the tumor tissue were significantly lower in patients with the MICA rs2596542 A allele than in those with the GG genotype (24.7 ± 15.1% vs. 41.5 ± 23.4%, P < 0.001).

### Factors associated with MICA expression in the tumor tissue and peritumoral tissue

As shown in Table 2, the MICA expression level in the peritumoral tissue did not differ between patients with different characteristics or cancer status. However, patients who carried the MICA rs2596542 A allele had significantly lower MICA expression levels in their tumor tissue compared with those with the GG genotype. Linear regression analysis revealed that the only factor correlated with MICA expression in the tumor tissue was the carriage of the MICA rs2596542 A allele (β: −0.396; 95% confidence intervals [CI]: −22.744–10.991; P < 0.001).

**Table 2:** MICA expression of the tumor and non-tumor part in patients with different characteristics.

Variable	Tumor part		Non-tumor part	
	%	P value	%	P value
Gender		0.12		0.92
Male	33.4 ± 20.5		30.3 ± 17.0	
Female	32.2 ± 24.0		30.6 ± 16.9	
Age		0.39		0.17
<65 years	31.8 ± 21.6		28.8 ± 16.5	
≥65 years	34.5 ± 21.2		32.2 ± 17.3	
BMI		0.13		0.74
<27 kg/m^2^	31.8 ± 20.4		30.6 ± 17.1	
≥27 kg/m^2^	37.3 ± 24.2		29.7 ± 16.6	
MICA rs2596542 genotype*		<0.001		0.39
A allele	24.7 ± 15.1		31.5 ± 16.9	
GG genotype	41.5 ± 23.4		29.4 ± 17.0	
Etiology		0.20		0.59
HBV	32.5 ± 21.1		29.5 ± 14.3	
HCV	27.9 ± 19.2		31.0 ± 18.5	
AFP		0.42		0.34
<20 ng/mL	34.2 ± 21.9		29.5 ± 17.1	
≥20 ng/mL	31.7 ± 20.4		31.9 ± 16.6	
Fibrotic stages		0.42		0.74
Non-cirrhosis	32.2 ± 20.2		30.1 ± 16.7	
Cirrhosis	34.9 ± 23.9		31.0 ± 17.5	
Child-Pugh score		0.33		0.11
A	32.5 ± 21.2		29.9 ± 16.4	
B	37.5 ± 22.0		37.6 ± 19.2	
BCLC stage		0.78		0.26
0/A	33.3 ± 22.3		31.2 ± 16.7	
B	32.4 ± 19.8		28.3 ± 17.0	
TMN stage		0.82		0.98
1/2	32.9 ± 22.1		30.4 ± 17.0	
3/4	33.8 ± 17.8		30.3 ± 16.7	
Tumor size		0.70		0.83
<5 cm	33.5 ± 22.1		30.2 ± 17.1	
>5 cm	32.3 ± 20.3		30.8 ± 16.7	
Tumor number		0.18		0.92
Single	32.1 ± 21.1		30.5 ± 17.3	
Multiple	37.8 ± 22.6		30.1 ± 15.3	
HCC differentiation^†^		0.82		0.86
Well/moderate	33.3 ± 21.9		28.7 ± 17.2	
Poor	34.3 ± 21.9		28.1 ± 14.7	

Note: BMI: body mass index.HBV: hepatitis B virus. HCV: hepatitis C virus. MICA: MHC class I chain-related A. BCLC: Barcelona Clinic Liver Cancer. HCC: hepatocellular carcinoma. ^*^6 patients had indeterminate single nucleotide polymorphism. ^†^Data available in 119 patients.

The best cut-off value of MICA expression levels to differentiate between patients with different MICA genetic variants in their tumor tissue was 30% (AUC of ROC 0.712, P < 0.001). The proportion of A allele carriage was significantly lower in patients with high MICA (≥30%) expression compared to those with low MICA expression (<30%) (27.1% vs 70.3%, P < 0.001). Logistic regression analysis revealed that the carriage of the MICA rs2596542 A allele was the only factor associated with low MICA expression in the tumor (odds ratio/CI: 0.16/0.08–0.30, P < 0.001) (Table 3).

**Table 3:** Factors associated with high MICA expression in the cancer.

	High MICA (n = 99)	Low MICA (n = 94)	P value	OR	C.I.	P value
Age (years, mean ± SD)	63.2 ± 12.1	62.3 ± 10.9	0.61			
Male gender, n (%)	79 (79.8)	65 (69.1)	0.09			
BMI (kg/m^2^, mean ± SD)	24.6 ± 3.2	25.1 ± 5.3	0.41			
AST (IU/L, mean ± SD)	56 ± 36	55 ± 40	0.92			
ALT (IU/L, mean ± SD)	48 ± 34	54 ± 45	0.29			
α-fetoprotein >20 ng/mL, n (%)	42 (42.4)	41 (43.6)	0.71			
Fibrosis stage 3–4, n (%)	54 (54.5)	50 (53.2)	0.85			
HBV/HCV, n	44/20	43/31	0.20			
Child-Pugh score A, n (%)	81 (81.8)	82 (91.1)	0.63			
MICA rs2596542 A allele, n (%)*	26 (27.1)	64 (70.3)	<0.001	0.16	0.08–0.30	<0.001
BCLC stage 0-A, n (%)	59 (59.6)	58 (61.7)	0.82			
TMN stage 1–2, n (%)	21 (21.2)	13 (13.8)	0.18			
Tumor size >5 cm, n (%)	34 (34.3)	36 (38.3)	0.57			
Tumor number >2, n (%)	19 (19.2)	13 (13.8)	0.32			
Poor differentiated HCC^†^	27 (42.9)	21 (37.5)	0.55			

Note: high MICA expression: >30%. MICA: membrane MHC class I chain-related A. BMI: body mass index. AST: aspartate aminotransferase. ALT: alanine aminotransferase. HCC: hepatocellular carcinoma. HBV: hepatitis B virus. HCV: hepatitis C virus. BCLC: Barcelona Clinic Liver Cancer. ^*^6 patients had indeterminate single nucleotide polymorphism. ^†^Data available in 119 patients. OR: odds ratio. CI: confidence intervals.

### Association of tumor MICA expression with HCC recurrence

**Figure 3:**
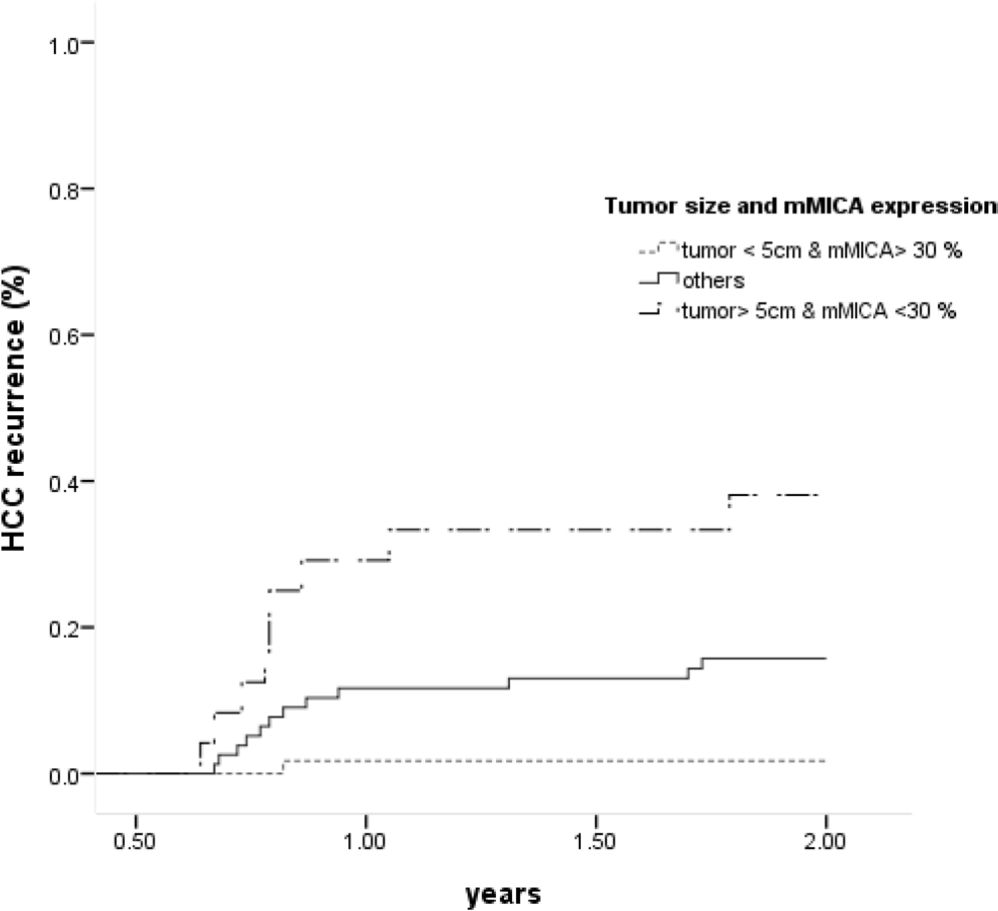
Kaplan–Meier analysis of HCC recurrence in patients with different risk factors including tumor size and cellular MICA expression. MICA: MHC class I chain-related A protein.

Fifty-one (26.4%) patients experienced HCC recurrence after surgery, with a mean follow-up period of 3.3 years (inter-quartile range: 0.8–4.8 years). The majority (n = 45, 88.2%) of the recurrent HCC occurred within 2 years of the surgical resection. We further analyzed the factors predictive of early HCC recurrence in the patient cohort. After excluding 22 patients who experienced HCC recurrence within six months after surgery, the patients with recurrent HCC had lower tumor MICA expression levels, more advanced TMN stages, BCLC stages and more tumors >5 cm (Table 4). Compared to patients without HCC recurrence, those with early HCC recurrence had significantly lower tumor MICA expression levels (24.0 ± 19.8% vs. 34.2 ± 21.8%, P = 0.03). Cox regression analysis revealed that the factors independently predictive of HCC recurrence included low MICA expression (hazard ratio [HR]/CI: 2.77/1.07–7.14, P = 0.035) and tumor size (HR/CI: 5.22/2.11–12.96, P < 0.001 (Table 4). During the 2-year follow-up period, the recurrence rate was 1.7% (1/58), 14.8% (12/81) and 28.1% (9/32) in patients who possessed no risk factors, one risk factor and both risk factors (tumor >5 cm and MICA expression <30%), respectively (P < 0.001) (Fig. 3). Compared to patients with tumors <5 cm and MICA expression >30%, patients who carried one or both risk factors had HCC HRs of 9.76 (C.I. 1.27–75.03, P = 0.03) and 27.30 (C.I. 3.46–215.6, P = 0.002), respectively. The risk of HCC recurrence did not differ between patients with “tumor <5 cm but MICA < 30%” and”tumor >5 cm but MICA > 30%” (P = 0.13).

**Table 4:** Factors associated with HCC recurrence.

	HCC recurrence (+) (n = 22)	HCC recurrence (−) (n = 149)	P value	HR	C.I.	P value
Age (years, mean ± SD)	61.9 ± 11.9	62.7 ± 11.4	0.76			
Male gender, n (%)	20 (90.9)	110 (73.8)	0.09			
BMI (kg/m^2^, mean ± SD)	23.7 ± 3.6	25.0 ± 4.6	0.22			
AST (IU/L, mean ± SD)	61 ± 41	55 ± 37	0.49			
ALT (IU/L, mean ± SD)	61 ± 43	51 ± 40	0.29			
α-fetoprotein >20 ng/mL, n (%)	9 (42.9)	62 (42.8)	0.84			
Fibrosis stage 3–4, n (%)	12 (54.5)	80 (53.7)	1			
Child-Pugh score A, n (%)	18 (85.7)	128 (90.1)	0.45			
MICA rs2596542 A allele, n (%)	12 (54.5)	65 (45.5)	0.30			
Tumor MICA expression* <30%, n (%)	15 (68.2)	71 (47.7)	0.04	2.77	1.07–7.14	0.035
BCLC stage 0-A, n (%)	7 (31.8)	101 (67.8)	<0.001			
TMN stage 1, n (%)	3 (13.6)	60 (40.3)	0.01			
Tumor size >5 cm, n (%)	15 (68.2)	44 (29.5)	<0.001	5.22	2.11–12.96	<0.001
Tumor number >2, n (%)	5 (22.7)	22 (14.8)	0.32			

Note: MICA: MHC class I chain-related A. BMI: body mass index. AST: aspartate aminotransferase. ALT: alanine aminotransferase. BCLC: Barcelona Clinic Liver Cancer. HR: hazard ratio. CI: confidence intervals. *Tumor part.

## Discussion

To our knowledge, this is the largest clinical study that has addressed the issue of the expression of membrane-bound MICA in HCC patients with different MICA genetic variants and the corresponding cancer recurrence outcomes. The MICA expression in the tumor tissue varied among HCC patients. Patient characteristics, including liver disease severity and HCC etiology, were not associated with MICA expression, and MICA expression was not associated with the cancer stages. We demonstrated that the MICA SNP was the only determinant of MICA expression in cancer tissues. The carriage of the MICA rs2596542 A allele was independently associated with low MICA expression in the tumor tissue but not in the peritumoral tissue. Interestingly, we identified that the MICA expression in the tumor tissue independently predicted the post-operative patient outcome. Patients with low MICA expression in their tumor tissue were at greater risk of HCC recurrence.

Circulating MICA is secreted by stressed cells. The abundant production of soluble MICA down-regulates and further suppresses the MICA-NKG2D-NK axis. As a result, the relationship between high levels of circulating MICA and oncogenesis has been well established^7,9–11,18–20^. By contrast, the carriage of the MICA rs2596542 A allele has been shown to result in the production of low levels of soluble MICA^7,9,10,14^. A GWAS study demonstrated that the carriage of the MICA rs2596542 A allele was associated with hepatitis C virus-related HCC in a Japanese cohort^7^. We recently replicated and extended that finding in Taiwanese chronic hepatitis C patients who failed antiviral therapy in a longitudinal follow-up cohort^9^. The association of the carriage of the A allele with low sMICA levels seemed to conflict with the proposal that HCC patients may have high sMICA production. A pathophysiological mechanism was postulated whereby A allele carriers may also have low membrane-bound MICA, which would attenuate the NKG2D engagement and consequently the innate immune recognition^7,8^. There is evidence of this in some fundamental studies. For example, MICA may not be detected in hepatocytes from normal liver tissue or liver tissue in the early stage of chronic hepatitis^11^. HBV-mediated disruption of MICA has also been observed in Hep G2.2.15 cells^21^. Notably, the expression of cellular MICA in patients with different MICA genetic variants has rarely been confirmed in clinical cohorts. The current study clearly demonstrated that MICA expression in tumor tissue was lower in MICA rs2596542 A allele carriers compared to patients with the GG genotype. The paucity of cellular MICA may prompt immune surveillance escape by the cancer cells, which supports the results of previous genetic studies.

Among patients with HCC, the link between MICA expression and the cancer pattern including prognosis is unclear. It has been suggested that patients with high soluble MICA have a higher proportion of vascular invasion and poorer survival^10^. We previously demonstrated that CHC patients with high sMICA levels are more likely to experience HCC recurrence if they fail antiviral therapy^22^. Kohga *et al*. showed that patients with high-grade (TMN stage III/IV) HCC had significantly higher levels of sMICA than did patients without HCC or with low-grade (TMN stage I/II) HCC^11^. Due to the relatively poor accessibility of HCC tissue samples, the relationship between cellular MICA levels and the clinical presentation of HCC has rarely been discussed. We noticed that the expression level of MICA in the tumor tissue was not associated with cancer behavior or stages at the time of surgery on a cross-sectional basis. However, we extended the observation to the post-operative follow-up period. We identified that tumor size may determine early HCC recurrence, which was in line with previous report^17^. Imperatively, we demonstrated that low MICA levels in the cancer tissue may play a critical biological role. Patients who had low MICA expression had a nearly 3-fold greater risk of recurrence compared to their counterparts. Poor immune recognition of cancer cells due to suboptimal MICA surveillance may contribute to that increased risk of recurrence^8^. This finding echoed the previous observation that dysregulation of NKG2D ligand, UL16-binding protein, on cancer cells may predict early HCC recurrence^23^. Importantly, a large tumor size and low MICA expression synergistically increased the risk of early HCC recurrence. The risk of HCC recurrence increased up to 27-fold in patients who possessed both unfavorable anatomic and biological characteristics in contrast to those without any risk factors. The observation that A allele was not associated with HCC recurrence in the study was not contradictory. This indicated that the genotypic difference at the nucleotide level would not directly drive the clinical presentation of early HCC recurrence. By contrast, the low cellular MICA expression in the phenotypic protein level had more tremendous impact on HCC recurrence. Further studies with larger sample size and longer postoperative follow-up observation are warranted to calcify the association of the genetic variants with late recurrence.

The current study was limited by the failure to compare the results with non-HCC controls and the current semi-quantitative immunohistochemistry stain could not precisely define the location of cellular MICA. This was a proof of concept study, and we aimed to confirm the MICA expression levels in HCC patients who possessed different genetic variants. The results strengthen the postulated pathophysiological mechanism whereby low cellular MICA expression increases the risk of HCC. Notably, the locus may have reciprocal results across different ethnicities, which attributed to distinct genetic backgrounds and environmental pressures^8^. In conclusion, the carriage of the MICA rs2596542 A allele was independently associated with low MICA expression in the tumor tissue. Low MICA expression in the cancer tissue predicts early HCC recurrence after curative treatment. Further studies with patients of different ethnicities are warranted to validate our finding.
